# Prenatal diagnosis of two de novo 4q35-qter deletions characterized by array-CGH

**DOI:** 10.1186/1755-8166-6-47

**Published:** 2013-10-31

**Authors:** Emmanouil Manolakos, Konstantinos Kefalas, Annalisa Vetro, Eirini Oikonomidou, George Daskalakis, Natasa Psara, Elisa Siomou, Elena Papageorgiou, Eirini Sevastopoulou, Anastasia Konstantinidou, Nikolaos Vrachnis, Loretta Thomaidis, Orsetta Zuffardi, Ioannis Papoulidis

**Affiliations:** 1 Eurogenetica, Laboratory of Genetics, Athens-Thessaloniki, Greece; 2 Bioiatriki S.A., Laboratory of Genetics, Athens, Greece; 3 Biotechnology Research Laboratories, Fondazione IRCCS Policlinico San Matteo, Pavia, Italy; 4 1st Department of Obstetrics & Gynecology, University of Athens, Athens, Greece; 5 Fetal Medicine Care, Thessaloniki, Greece; 6 1st Department of Pathology, University of Athens, Athens, Greece; 7 2nd Department of Obstetrics and Gynaecology, Aretaieion Hospital, University of Athens, Athens, Greece; 8 1st Department of Pediatrics, Aglaia Kyriakou Children’s Hospital, Athens, Greece; 9 Department of Molecular Medicine, University of Pavia, Pavia, Italy; 10 Cattedra di Genetica Medica, Universita di Cagliari, Cagliari, Italy

**Keywords:** 4q- syndrome, Array-CGH, Prenatal diagnosis, Deletion 4q35.1

## Abstract

**Background:**

The 4q- syndrome is a well known genetic condition caused by a partial terminal or interstitial deletion in the long arm of chromosome 4. The great variability in the extent of these deletions and the possible contribution of additional genetic rearrangements, such as unbalanced translocations, lead to a wide spectrum of clinical manifestations. The majority of reports of 4q- cases are associated with large deletions identified by conventional chromosome analysis; however, the widespread clinical use of novel molecular techniques such as array comparative genomic hybridization (a-CGH) has increased the detection rate of submicroscopic chromosomal aberrations associated with 4q- phenotype.

**Results:**

Herein we report two prenatal cases of 4qter deletions which presented the first with no sonographic findings and the second with brain ventriculomegaly combined with oligohydramnios. Standard karyotyping demonstrated a deletion at band q35.1 of chromosome 4 in both cases. The application of a-CGH confirmed the diagnosis and offered a precise characterization of the genetic defect.

**Conclusions:**

We provide a review of the currently available literature on the prenatal diagnostic approach of 4q- syndrome and we compare our results with other published cases. Our data suggest that the identification and the precise molecular characterization of new cases with 4q- syndrome will contribute in elucidating the genetic spectrum of this disorder.

## Background

The 4q deletion syndrome is a rare chromosomal disorder caused by a partial deletion of the long arm of chromosome 4, with an estimated incidence of 1 in 50,000-100,000 [1,2]. Since the first description of a deletion at the terminal region of the long arm of chromosome 4 by Ockey et al. in 1967, more than 150 literature cases have been reviewed and delineated as 4q- syndrome [3], most of them diagnosed postnatally. The majority of deletions are de novo but approximately 14% of cases result from unbalanced segregation of parental reciprocal traslocations [2]. Male to female ratio is approximately equal 48:53. The 4q- cases can be further classified depending on the exact chromosome breakpoints.

Common phenotypic characteristics of 4q- syndrome are mild dysmorphic features, craniofacial, digital, skeletal, gastro-intestinal, cardiac and urogenital anomalies, while most probands are presented with variable developmental delay and growth deficiency [1,4-7].

In literature, the majority of postnatally diagnosed cases are associated with large 4q deletions identified by conventional chromosome analysis. However, nowadays the widespread clinical use of novel high-resolution molecular techniques such as array comparative genomic hybridization (a-CGH), has increased the detection rate of submicroscopic chromosomal aberrations that could also lead to a 4q- phenotype. In this report, we applied genome-wide array-CGH to investigate further, two cases detected prenatally with distal deletions of chromosome 4q. A detailed review of the current literature on prenatal diagnosis of 4q- syndrome is also provided.

## Results

### Clinical reports

#### Case 1

A 36-year-old primigravida was referred to our clinic at 19 weeks of gestation due to IVF and parental anxiety. The family history was unremarkable and the first-trimester screening test for chromosomal aneuploidy was normal. No fetal malformation was present in ultrasound scan. Amniocentesis was performed and karyotype analysis led to the diagnosis of a 4qter deletion which was further confirmed by a-CGH. After genetic counselling, termination of pregnancy was performed at parent’s request at 22 weeks of gestation. A male neonate was delivered vaginally after medical induction with prostaglandins. Necropsy showed a male fetus with macrocephaly, depressed nasal bridge, micrognathia, carpal flexion, and clinodactyly.

#### Case 2

A 27-year-old primigravida was referred to our clinic at 22 weeks gestation for genetic counselling due to presence of brain ventriculomegaly and oligohydramnios in the detailed second trimester anomaly ultrasound scan. The first trimester screening test for fetal aneuploides was normal and the couple was healthy, no consanguineous, with unremarkable medical history. Invasive testing was offered and amniocentesis was performed. Karyotype analysis led to the diagnosis of a 4qter deletion and array-CGH analysis confirmed with high precision a 8.0 Mb terminal 4q deletion, in the area 4q35.1-qter. After extensive counselling, the family decided to terminate the pregnancy and agreed to an autopsy of the fetus. A male stillborn neonate was delivered at 24 weeks after medical induction with prostaglandins. Fetal autopsy revealed a fetus which was appropriate for age, weighing 692 g and measuring 22.5 cm in CRL and 50 mm in foot length. Dysmorphic craniofacial features included macrocephaly, hypertelorism, beaked nose, depressed nasal bridge, increased hair growth with low frontal and cervical hairline and hair extending onto the lateral cheeks. Increased hair growth was also noted on the back and arms. The neck was broad and short. Limb deformities included camptodactyly, overlapping fingers, and “rocker bottom” feet with prominent heels. External genitalia were of normal male. On dissection, the anterior fontanelle was distended; measuring 4.0 × 2.8 cm. Neuropathological examination was not feasible due to brain autolysis. The diaphragm was membranous. Organ weights were appropriate for age. Microscopy showed increased erythropoiesis in the liver. No further gross or histologic abnormalities were noted. According to external maceration and autolytic lesions of fetal tissues, intrauterine fetal death was approximately dated 24 h before delivery.

The placenta, 113 g of weight, was very small for gestational age, with a partially extrachorial configuration and histologic findings of maternal hypoperfusion and decidual vasculopathy. The umbilical cord inserted peripherally.

### Cytogenetic and molecular cytogenetic analysis

Cytogenetic analysis was performed on cultured amniocytes and revealed a male karyotype with a terminal deletion of the long arm of one chromosome 4 in both cases. The karyotype was described as 46,XY,del(4)(q35.1) according to ISCN 2013. Parental karyotyping was found to be normal (data not shown) in both cases.

**Figure 1:**
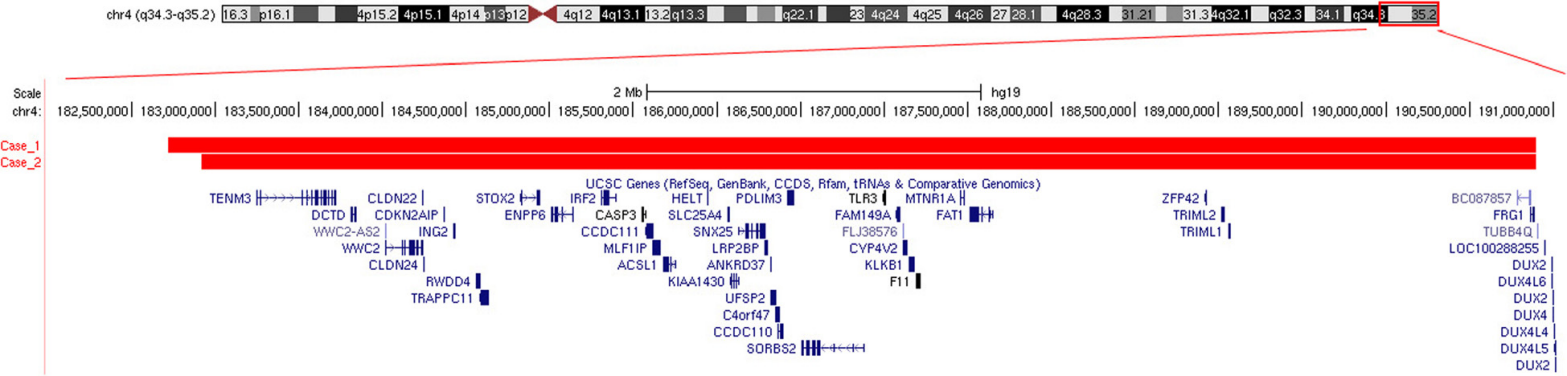
**UCSC genome browser (GRCh37/hg19) illustration of the terminal region of the long arm of chromosome 4 from base pairs 182,500,000 in 4q34.3 to 191,000,000 in 5p13.3.** The region is indicated by the rectangle on the idiogram of chromosome 4. The corresponding deleted segments in our two cases are highlighted in red, while the gene content is also displayed. The overall size of the deletion was about 8 Mb in both cases.

The two aberrations were further investigated using array-CGH technique. In case 1, a-CGH analysis detected a 8.18 Mb deletion which proximal and distal breakpoints fell between 182,717,805 and 190,896,815 bp (last oligonucleotide probe available on platform), respectively, extending from 4q35.1 to the subtelomeric region of 4q35.2 (Figure 1). In case 2 the chromosome 4 abnormality was further delineated as a 8 Mb deletion which proximal and distal breakpoints fell between 182,920,816 and 190,896,815 bp (last oligonucleotide probe available on platform), respectively (Figure 1). The positions of oligomers refer to the Human Genome February 2009 (versions GRCh37, hg19) assembly.

## Discussion

Routine prenatal chromosome analysis identifies rarely chromosomal anomalies under 5 Mb, whilst array-CGH can detect small chromosomal imbalances anywhere in the genome [8]. Moreover, array-CGH has the potential to define exactly the size of the deletion and its gene content, at a much higher resolution than karyotyping, thus making easier a genotype-phenotype correlation in many cases. Recent studies have demonstrated that careful implementation of high resolution array testing would benefit at least 2-10% of obstetric patients with abnormal ultrasound findings and a normal karyotype, by revealing genetic rearrangements with clinical significance [9,10]. Furthermore, genome-wide array is now considered essential for the prenatally characterization of findings with elusive clinical significance, such as apparently balanced de novo chromosome rearrangements, supernumerary marker chromosomes of unknown origin and structural variations not classified as already known benign polymorphisms [11]. However, conventional karyotyping can detect balanced rearrangements such as reciprocal and Robertsonian translocations, inversions and balanced insertions. These alterations would have gone undetected by microarray analysis, therefore not enabling the family to be aware of the potential for unbalanced offspring in the future [11]. The majority of prenatally diagnosed cases of 4q- syndrome reported in the literature are delineated by conventional cytogenetic analysis, but during the last decade the availability of new technologies especially a-CGH, have enabled a more precise description of the molecular mechanisms that can account for the 4q- phenotype [12].

We report two prenatal cases of 4q- syndrome. The first was presented with normal ultrasound findings and prenatal testing was offered due to increased maternal age and parental anxiety. The second was referred to our laboratory due to presence of brain ventriculomegaly and oligohydramnios. After invasive testing, conventional cytogenetic investigation led to diagnosis of a terminal 4q deletion at region q35.1 in both cases. In addition, molecular analysis of cultured amniocytes with a-CGH further defined the precise breakpoints of the two aberrations (Figure 1), revealing two terminal deletions approximately 8 Mb in size respectively. Interestingly, only a few prenatally diagnosed cases of 4q- syndrome have been reported in the bibliography. In Table 1, we summarize the clinical features of six prenatal cases (included ours) with pure, terminal deletions of the segment 4q33-qter or a part of it.

**Table 1:** Clinical findings in prenatally diagnosed cases with overlapping chromosome 4q terminal deletions

**Studies**	**Russel et al. 2008**[13]	**Rossi et al. 2009 **[14] **Case 1**	**Rossi et al. 2009 **[14] **Case 2**	**Strehle et al. 2012 **[3] **Case 1**	**Curent** **Case 1**	**Current** **Case 2**
**Deletion**	4q33-qter	4q34.1-qter	4q34.1-qter	4q33-qter	4q35.1-qter	4q35.1-qter
**Size**	-	12.6	16.5	-	8.2	8.0
**Start Seq.**		178.5	174.6	-	182.7	182.9
**End Seq.**		191.1	191.1	-	190.9	190.9
**Gestation age**	32 w	13 w	-	13 w	19 w	22 w
**Sex (M/F)**	F	F	F	F	F	M
**Maternal age**	31 y	33 y	n.d.	43 y	36 y	28 y
**Pregnancy length**	34 wks	term	term	TOP at 18+5 w	TOP at 22 w	TOP at 24 w
**Birth weight (g)**	2,660	3,490	5^th^ percentile	-	-	692
**Birth length (cm)**	45	53.5	5^th^ percentile	-	-	50 mm
**Birth OFC (cm)**	n.d.	36	-	-	-	-
**Growth retardation**	Yes	-	-	No	No	No
**Mental retardation**	-	LD	Yes	-	-	-
**Cranial/Facial abnormalities**	Yes	Yes	Yes	Yes	Yes	Yes
**Microcephaly**	No	-	-	-	No	No
**Macrocephaly**	No	-	-	-	Yes	Yes
**Forehead**	High	-	-	-	-	-
**Eyes**	-	Iris coloboma	Myopia	-	-	Hypertelorism
**Nose**	Small with narrow bridge and anteverted nares	-	Short nose	-	Depressed nasal bridge	Beaked
**Micrognathia**	Yes	Yes	-	-	No	No
**Heart**	VSD	VSD, PDA	-	Normal	No	No
**Limb abnormalities**	Yes	-	-	-	yes	Yes
**Hands**	4^th^ and 5^th^ fingers bilaterally	-	Clinodactyly of the left and right fifth toes	-	Clinodactyly	Campodactyly
**Feet**	Short	-	-	-	-	-

-, absence; n.d, not determined; TOP, termination of pregnancy; VSD, ventricular septal defect; PDA, patent ductus arteriosus; LD, learning disabilities in carrier mother.

4q deletion syndrome is a distinct congenital malformation syndrome associated with various clinical findings affecting multiple systems and organs, including facial and digital dysmorphology, Pierre Robin sequence, developmental delay, autistic spectrum disorder, and abnormalities of the cardiovascular, musculoskeletal and gastrointestinal systems [12]. However, several reports of concomitant 4q- and other structural chromosomal aberrations, as a result of de novo or inherited unbalanced translocations involving a deletion on chromosome 4 and a partial trisomy for another chromosome arm, display complex phenotypes and confuse some of the correlations [2].

About 40 genes with either known or unknown functions are included in the deleted 4q35.1-qter region (182.7 to 190.9 Mb). More specifically these genes encode enzymes (*ENPP6, F11, CYP4V2, DCTD, KLKB1, ACSL1*), signal receptors and transducers (*MTNR1A, SORBS2, FAT1*), structural proteins (*MLF1IP, PDLIM3, CLDN22*), one protein member of caspase family (*CASP3*), one transporter in mitochondrial membrane (*SLC25A4*), two transcription factors (*HELT, IRF-2*) and two proteins implicated in innate immunity (*TLR-3, IRF-2*). Four of these genes are reported as disease genes in OMIM database. Specifically *SLC25A4* is connected with progressive external ophthalmoplegia and familial hypertrophic cardiomyopathy, *TLR-3* with susceptibility or resistance to some kinds of infections, *F11* with deficiency in coagulation factor XI and *CYP4V2* with Bietti crystalline corneoretinal dystrophy. However, none of these, alone, can account for the 4q- syndrome phenotype. After taking into consideration all the published cases and two of their own, Rossi et al., suggested a 3 Mb interval of terminal 4qter, which possibly contains haploinsufficient genes and is responsible for 4q- syndrome [14]. This region extends from 186 to 189 Mb and the candidate genes are *HELT, LRP2BP, SORBS2* and *FAT1*. It is worth taking into consideration that the same region is included in both deletions here described. Recently, Strehle et al. published a genotype-phenotype analysis of 20 individuals with 4q- syndrome and variable extent of deletions, which were assessed by a-CGH technique. The author suggested that the critical region for the syndrome resides in 4q35.1, implicating gene *SORBS2* for orofacial clefts and congenital heart defects [12]. *SORBS2* encodes a protein containing N-terminal sorbin and a C-terminal SH3 domain, acting as signal transducer in epithelial and cardiac muscle cells, possibly by linking Abl family kinases and the actin cytoskeleton.

Interestingly, although *SORBS2* gene is deleted in our two described cases, however no cleft palate was present. Strehle et al. highlight that a specific gene responsible for clefts or influencing palate development should reside in 4q33-4q35.1, but it could not be exactly located yet [12]. The author describes a patient (case 20) with a minimal deletion in 4q35.1 (186.7-187.2 Mb) who is presented with cleft palate. However, he also reports other cases with larger deletions in the same area, who do not display the same feature (cases 14, 16, 18, 19) [12]. Moreover, Rossi et al. after considering their two cases and the current literature, suggested that the region responsible for cleft palate should reside between 174 to 178 Mb in 4q34.1-4q34.2 [14]. Taking in account all these contradictory observations, we can hypothesize that other genes or genetic factors could modify the phenotype in each particular case, thus rendering the efforts of determining the responsible gene(s) a challenging task.

## Conclusions

In conclusion 4q- syndrome is associated with facial dysmorphism and/or other major malformations such as skeletal abnormalities, midline fusion defects and renal hypoplasia. These findings may be indicative of a 4q- case and should trigger cytogenetic investigation that will eventually lead to an accurate diagnosis. In our cases, we applied array-CGH analysis in two 4q- fetuses with cytogenetically visible deletions so as to confirm that they were pure distal deletions, to define their extent at molecular level and to establish a firm diagnosis. Standard chromosome analysis that reveals a normal karyotype, may lead to false interpretations and further investigation with high-resolution techniques such as array-CGH is nowadays strongly recommended, particularly in case of discordance between prenatal ultrasound findings and normal karyotype. In the future, the implementation of this technique in the routine practice of prenatal diagnosis will improve the diagnostic yield in pregnancies with abnormal ultrasound findings and particularly to 4q-, it will enable a more precise estimation of the true incidence of the syndrome and shall advance our knowledge regarding the genotype-phenotype correlations.

## Methods

Amniotic fluid was collected from case 1 and case 2 at 19 and 22 weeks of gestation, respectively. Cytogenetic analysis was performed on cultured amniocytes by G-banding according to standard procedures. At least 20 metaphases from two different cultures were analyzed per case. Chromosome analysis of parental blood samples was performed using GTG-banding techniques on stimulated blood lymphocytes at 550–600 band resolution.

Array-CGH was performed on DNA from cultured amniocytes to characterize the extent of the deletions using an 80 kb resolution array (kit 60 K). Molecular karyotyping was carried out through oligonucleotide array-CGH platforms (Agilent Technologies, Santa Clara, CA) as described elsewhere [15]. The positions of oligomers refer to the Human Genome February 2009 (versions GRCh37, hg19) assembly.

## Competing interests

The authors declare that they have no competing interests.

## Authors’ contributions

EM, KK, AK and LT wrote the manuscript. GD, NP, NV and ES referred the patients for study. AK performed the autopsies. EM, EP and EO performed the cytogenetic analysis. AV and OZ signed out the molecular cytogenetic results. IP coordinated the study. All authors have read and approved the manuscript.
